# Persisting Effects of Ayahuasca on Empathy, Creative Thinking, Decentering, Personality, and Well-Being

**DOI:** 10.3389/fphar.2021.721537

**Published:** 2021-10-01

**Authors:** Maggie K. Kiraga, Natasha L. Mason, Malin V. Uthaug, Kim I.M. van Oorsouw, Stefan W. Toennes, Johannes G. Ramaekers, Kim P. C. Kuypers

**Affiliations:** ^1^ Department of Neuropsychology and Psychopharmacology, Faculty of Psychology and Neuroscience, Maastricht University, Maastricht, Netherlands; ^2^ Department of Clinical Sciences, Faculty of Psychology and Neuroscience, Maastricht University, Maastricht, Netherlands; ^3^ Institute of Legal Medicine, University of Frankfurt, Frankfurt, Germany

**Keywords:** ayahuasca ceremony, persisting effects, empathy, creativity, well-being, decentering

## Abstract

**Background:** Naturalistic and placebo-controlled studies have suggested that ayahuasca, a traditional Amazonian beverage, could be helpful in the treatment of psychopathologies like depression and anxiety disorders by changing otherwise disturbed cognitive and emotional processes. To better understand its full therapeutic potential, one way is to study the effects on processes like flexible thinking, empathy, and well-being, which are normally compromised in stress-related psychopathologies.

**Materials and Methods:** Volunteers attending ayahuasca ceremonies were asked to complete a test battery at three separate occasions: baseline, the morning after, and 1 week after the ceremony. We included the constructs of creative thinking (measured by Picture Concept Test), empathy (Multifaceted Empathy Test), satisfaction with life (Satisfaction with Life Scale), decentering (Experiences Questionnaire), and personality (Big Five Inventory) into the test battery. Additionally, the psychedelic experience was quantified with the Persisting Effects Questionnaire, the Ego Dissolution Scale, and Visual Analogue Scales.

**Results:** In total, 43 attendees (males = 22; females = 21) completed parts of the baseline assessment, 20 (males = 12; females = 8) completed assessments in the morning after the ceremony, and 19 (males = 14; females = 5) completed assessments at the 1-week follow-up. At one and 7 days post-ceremony, cognitive empathy, satisfaction with life, and decentering increased, while divergent thinking (*Fluency* corrected for *Originality*) decreased, when compared to baseline. Implicit emotional empathy increased at 1-week follow-up, whereas ratings of the trait *neuroticism* decreased.

**Conclusion:** The study suggests that a single ingestion of ayahuasca in a social setting is associated with enhancement of subjective well-being, an enhanced ability to take an objective and non-judging stance towards the self (decentering), and the ability to correctly recognize emotions in others, compared to baseline, lasting up to 1 week post-ceremony. To understand the therapeutic potential related to these effects, further research with clinical populations is needed in which these effects can be assessed, including its link with therapeutic outcomes. Together, this will increase our understanding of the effectiveness and breadth of future therapeutic options.

## Introduction

Ayahuasca is a psychoactive decoction with a reported history of use for magical, ritual, and medicinal purposes by indigenous groups of the Amazon (Schultes 1986; Schmid 2012; Frecska et al., 2016). In addition to traditional historical practices, there has been an increase in the availability of the brew to non-Amazonian populations (Tupper 2008), with people reportedly using the substance to seek insights, personal growth, emotional healing, connection with nature, out of curiosity (Winkelman 2005; Uthaug et al., 2021).

Ayahuasca is made from the stalk of the vine *Banisteriopsis caapi*, a source of monoamine oxidase inhibitors (MAOIs), and the leaves of the *Psychotria viridis* shrub, a source of N,N-dimethyltryptamine (DMT; Palhano-Fontes et al., 2015). The combination of MAOIs and DMT is required to produce the psychedelic effects (Riba et al., 2003), including shifts in cognition, emotional lability, and visionary experiences (Shanon 2002).

Studies have shown that ayahuasca could be helpful in the treatment of psychopathologies (Barbosa et al., 2005; Palhano-Fontes et al., 2021; Sarris et al., 2021). To get a better understanding of ayahuasca’s full potential, one way is to study its effects on mental and cognitive processes that are generally compromised in stress-related psychopathologies, like flexible thinking, empathy, metacognitive awareness, and well-being (Tull and Roemer, 2007; Aldao and Nolen-Hoeksema, 2010; Parlar et al., 2014; Morrison et al., 2016; Mason et al., 2019; Mason and Kuypers 2021). Previously, it was demonstrated that divergent thinking (DT) of attendees of ayahuasca ceremonies was augmented during the altered state of consciousness compared to their baseline performance, while convergent thinking (CT) was decreased (Kuypers et al., 2016). Days to months after the self-administration of ayahuasca, individuals displayed enhanced flexible (divergent) and convergent thinking (Frecska et al., 2012; Uthaug et al., 2018; Uthaug et al., 2019). Evidence to date also points to the beneficial effects of the brew on self-reported levels of life satisfaction (Uthaug et al., 2018) and depressive symptoms (de Lima Osório et al., 2011; Palhano-Fontes et al., 2019; Sanches et al., 2016; van Oorsouw et al., 2021). Studies directly assessing ayahuasca’s effects on emotion regulation showed sub-acute improvements in emotional non-acceptance, emotional interference, and lack of control (Domínguez-Clavé et al., 2019) and lower scores on emotional dysregulation compared to the baseline that were sustained over the 1 year follow-up period (Bouso et al., 2012). Increments in implicit empathy (often referred to as “arousal”) were shown with attendees of ayahuasca group ceremonies who received ayahuasca vs. those who received placebo (Uthaug et al., 2021). Additionally, ingestion of ayahuasca was found to be beneficial for mindfulness-related capacities (Soler et al., 2016), levels of assertiveness and joy (Barbosa et al., 2005), anxiety reduction, optimism, and self-confidence (Da Silveira et al., 2005).

Despite the promising findings and the growing evidence suggesting a psychotherapeutic potential of the Amazonian decoction, there is still much knowledge to be gained about cognitive and psychological processes affected by ayahuasca, and the longevity of these effects. A naturalistic, observational study using a comprehensive test battery repeated over 1 week was set up with attendees of ayahuasca ceremonies to reproduce some of the well-known mood- and mindfulness-related benefits and expand the sparse findings on effects on empathy and personality. By administering the test battery on three separate occasions (baseline, the morning after, and 1 week after the ceremony), we sought to distinguish between the direct (sub-acute) and indirect (persisting) effects of the brew on a range of measures, including flexible thinking, empathy, and satisfaction with life. Based on the existing body of literature, we hypothesized that -during post-ceremonial measurements-participants would have greater creative and empathic abilities and higher ratings of life satisfaction and decentering, the ability to observe one’s thoughts and feelings in a detached manner (Soler et al., 2014). Additionally, given the reports on psychedelic-induced changes in personality traits (Bouso et al., 2012; Bouso et al., 2015; Nour et al., 2017; Johnstad 2021) and its understudied status in the context of ayahuasca ceremonies, a personality assessment was included. Our hypotheses were in line with the novel research suggesting psychedelics-induced increases in ratings of *openness* (Nour et al., 2017; Johnstad 2021) and decreases in *neuroticism* (Erritzoe et al., 2018).

## Materials and Methods

### Participants

Participants were volunteers attending ayahuasca retreats in the Netherlands, stemming from across four different locations, between 2017 and 2019. Attendees of those ceremonies were either invited to participate in the study on site, or contacted the researchers by email after hearing about the study through the retreat organizers. To participate in the study, volunteers had to be a minimum of 18 years old and proficient in English. Participants completed the test battery three times: at baseline, within 24 h after (hereafter referred to as sub-acute), and 7 days after the ayahuasca ceremony (hereafter referred to as follow-up). After being informed about the study and giving their consent, participants completed the baseline assessment in the week before they took part in an ayahuasca ceremony either online, or on-site. The next day, participants completed the sub-acute measurements on-site with the researcher. Seven days later, they received the final follow-up measurement online. The total amount of ayahuasca taken by each participant was recorded, and a sample of the ayahuasca was taken to determine the concentrations of alkaloids afterwards.

The study was conducted in accordance with the Declaration of Helsinki and subsequent amendments concerning research in humans and was approved by the Ethics Review Committee of Psychology and Neuroscience and Maastricht University (ERCPN-175-03-2017). Participation was voluntary and no incentives to participate were provided. All volunteers gave their written informed consent to participate. The research team was not involved in the screening, preparation, organization, administration, and supervision of the ayahuasca ceremonies that were visited.

### Study Procedure

#### Ayahuasca Ceremonies

The neoshamanic ceremony facilitators conducted personal intakes prior to participation in the ceremonies. These included screening for the individual’s physical and mental fitness to participate in the ceremony and the run through the preparatory instructions (e.g., to abstain from medications). Facilitators were not clinically trained to screen for health or mental disorders.

The general setting and organization of the ceremonies was similar throughout all of the retreats. Participants stayed in a large house together, hosted by at least two or more facilitators. Before ayahuasca ingestion, participants were acquainted with one another and were invited to take part in an “opening circle,” during which each participant was given the opportunity to share with the rest of the group any personal details that they felt comfortable with. The goal was to: 1) help the group get to know each other; 2) clarify motivations for the participation in the ceremony; 3) and discuss desired outcomes of the experience. Participants ingested ayahuasca in the evening hours, and after ingestion, participants generally stayed in one room lying on a mattress or floor mat with a blanket, pillow, bucket, bottle of water, and tissue box to the side. In the room, lights were dimmed, and herbs and tobacco were burned. The day following the ingestion, participants came back together to discuss their experience with each other and the facilitators.

That said, the ceremonies also differed regarding happenings before, during, and after ayahuasca intake. Specifically, depending on the organizer, the type of opening circle differed, in that organizers invested different amounts of time in discussing why individuals were present, what they expected, and also included activities for the attendees to get to know each other, like dancing, personal sharing, and games. During the ayahuasca ceremony, the type of music being played and the availability of musical instruments could differ. Some facilitators sung healing chants (icaros) during the ceremonies. Additionally, the retreats were of various lengths, with a minimum of 2 days (and one night of ingesting ayahuasca), and a maximum of 4 days (and two nights of drinking ayahuasca). Importantly, participants always completed their baseline measurement before they ingested ayahuasca, and their sub-acute measurement the day after their first ayahuasca ingestion. Generally, it has been shown that psychotropic effects of ayahuasca start between 30 and 60 min post-administration, reaching maximum intensity between 60 and 120 min, and can last up to 4 h after administration, although the exact time-window of the acute effects can vary strongly, depending on the amount taken and participant’s characteristics (Riba et al., 2001).

### Ayahuasca Sample

In total, four ayahuasca samples were obtained, one from each ceremony site. The alkaloid concentrations of the ayahuasca preparations were determined after dilution using high-performance liquid chromatography-electrospray ionization-time-of-flight mass spectrometry (LC-MS) which was calibrated with pure reference substances of N, N-dimethyltryptamine (DMT; Cerilliant, Round Rock TX, US), harmine, and harmaline (Aldrich Chemistry, St. Louis MO, US).

### Assessments

The assessments included a demographic section, the multifaceted empathy test (MET), the picture concept test (PCT), and six questionnaires: the Satisfaction with Life scale (SWLS), the Big Five inventory (BFI), the Experiences questionnaire (EQ), the Ego Dissolution Inventory (EDI), a 10 item visual analogue scale (VAS), and the Persisting Effects Questionnaire (PEQ). All the material was provided in English. Most of the measures were filled out three times, i.e., at baseline, sub-acute, and follow-up. Exceptions of this included the EDI and VAS, only filled out once, post-session, to assess the psychedelic experience in retrospect, the PEQ, which was only performed at the follow-up to assess persisting effects of the experience, and the BFI, which was only assessed at baseline and follow-up.

### Multifaceted Empathy Test

The Multifaceted Empathy Test (MET) provides behavioral indices of cognitive and affective aspects of empathy (Dziobek et al., 2008). It consists of 40 photos of people in complex emotional states, both negative, and positive (50% pictures of each valence). Cognitive empathy (CE) is evaluated based on a recognition task, in which participants had to select one word (out of four) best matching the emotional state expressed by the person in the picture. Given the complexity of depicted emotional states, the words were provided in three languages: English, Dutch, and German. The possible range of scores for CE is 0–40 (one point for each emotion recognized correctly). Emotional empathy (EE) assessed by this test can be divided into two subcategories: implicit and explicit. The former is estimated by participant’s ratings of ‘how aroused this picture made them feel’, while the latter by ratings of “how concerned they felt for the person in the picture”. Both ratings of EE are assessed on a scale from 1 to 9. The summed and valence-specific accuracy of the correctly recognized emotions and averaged and valence-specific ratings of arousal and concern were used as dependent variables. Previous validity and reliability analysis of the MET have shown to be in the good to highly satisfactory range (Dziobek et al., 2008).

### Picture Concept Test

The picture concept test (PCT) was used to assess creativity (Kuypers et al., 2016). The PCT consists of stimuli containing between 4 and 12 color pictures shown in two or three rows. The pictures were taken from the Wechsler Preschool, Primary Scale of Intelligence, and the Wechsler Intelligence Scale for Children. Participants were instructed to find an association between one of the pictures in each row, with instructions to provide one solution only, as there is one correct answer. The total number of correct answers serves as the dependent measure of convergent thinking.

To assess divergent thinking, participants were asked to provide as many alternative associations as possible between the colored pictures. This is the regular instruction included in measures of divergent thinking, and it is used to calculate several parameters, i.e., originality, fluency, and the ratio of both, which reflects the quality of divergent thinking as originality is corrected for quantity. Fluency is defined as the number of alternative associations. Originality is calculated by evaluating the originality of the alternative association relative to those provided by all other participants in a session. Alternative answers that were uniquely reported by a single participant received an originality score of 2. Answers that were shared with a single participant were valued as 1, and answers that were shared by three or more participants were rated zero. Mean originality (creativity) scores and ratio, originality scores weighed for fluency (originality/fluency), were used as measures of divergent thinking.

At the time of each measurement, a different (parallel) version of PCT was used to avoid learning effects. Each parallel version consisted of 17 stimuli, the participant was given 30 s per stimulus.

### Satisfaction with Life Scale

The Satisfaction With Life Scale (SWLS) has been developed to quantify the life satisfaction component of subjective well-being (Diener et al., 1985). The SWLS is five items long and includes sentences like I*n most ways, my life is close to my ideal*; *So far I have gotten the important things I want in life.* The participants were asked to rate every item on a seven-point Likert scale, ranging from 1 (*Strongly disagree*) to 7 (*Strongly agree*). The total score ranges from 5 to 35 and is obtained by summarizing the ratings given per each item. Higher scores indicate greater life satisfaction, with a score of 20 representing a neutral point on the scale. The original scale has high internal consistency (coefficient alpha ranging from 0.79 to 0.89) and good test-retest correlations (up to 0.84 with one-month interval; Diener et al., 1985).

### Big Five Inventory

The 44-item English version of the Big Five Inventory (BFI), developed by John et al. (1991) was used to measure personality traits. The scale was constructed to allow efficient and flexible assessment of the five personality dimensions: *extraversion, agreeableness, conscientiousness, neuroticism*, and *openness to experience*. Participants rated each BFI item on a 5-point scale ranging from 1 (*disagree strongly*) to 5 (*agree strongly*); Scale scores were computed by summing items associated with each trait. The five dimensions were measured by a different number of items. Overall, the scores range between 8 and 50 for each personality trait. The BFI scales have shown substantial internal consistency, retest reliability, and clear factor structure (John and Srivastava, 1999).

### Experiences Questionnaire–Decentering

The Experiences Questionnaire (EQ) is a self-report instrument that originally assessed decentering and rumination and was validated by Fresco and colleagues (2007). The 11-item EQ-Decentering (EQ-D) subscale was developed to quantify the ability to observe one’s thoughts and feelings in a detached manner. Participants rated items on a 5-point Likert-type scale (1 = *never* to 5 = *all the time*), assessing three facets: the ability to distinguish one’s self from one’s thoughts, the ability to not habitually react to one’s negative experiences, and the capacity for self-compassion (Fresco et al., 2007). The total score was calculated by calculating the mean of all 11 items and ranges from 1 to 5. The psychometric characteristics of the original scale have shown a robust structure for the decentering factor, with high internal reliability (Fresco et al., 2007).

### Ego Dissolution Inventory

The Ego Dissolution Inventory (EDI) is an eight-item self-report scale that assesses the participant’s experience of ego dissolution (Nour et al., 2016). In the present study, the original, English version was used to acquire a better understanding of the experiences the participants had about ego dissolution during the ayahuasca ceremony. For example “*I experienced a dissolution of my* “*self or ego*” and “*I felt at one with the Universe*”*.* The participants answered the scale with endpoints of either 0 (*No, not more than usual*) or 100 (*Yes, I experienced this completely/entirely*). The EDI was scored by calculating the mean of all items, with a higher total score indicating a stronger experience of ego dissolution. The scale has been shown to have excellent internal consistency (Nour et al., 2016).

### Visual Analogue Scale

The morning after the ceremony, participants were asked to retrospectively rate the intensity of various aspects of the acute ayahuasca experience using 10 visual analogue scales (VASs). These included 10 cm horizontal lines, with a bottom anchor of *not more than usual* and a top anchor of *much more than usual*. Example items were: These items have previously been shown to be sensitive to the acute effects of psychedelics (Carhart-Harris et al., 2012; Mason et al., 2019).

### Persisting Effects Questionnaire

The Persisting Effects Questionnaire (PEQ) is a 143-item long scale aiming to assess changes in attitudes, moods, behavior, and spiritual experience (Griffiths et al., 2006). Prior research found that PEQ is sensitive to the prolonged effects of psychedelics, occurring even a 1 year after the ingestion (Schmid and Liechti 2018). Due to time constraints, the current study used a shortened version of the scale (90 items), including five out of six main categories. Specifically, the following categories were tested: *attitudes about life* [Number of items (N) = 26]; *attitudes about self* (N = 22); *mood changes* (N = 18); *relationships* (N = 18); and *behavioral changes* (N = 2). The 86 items of these five categories were rated on a 6-point scale (ranging from 0 = *none* to 5 = *extreme*). The scores of the resulting 10 scales (positive and negative scales for each of five categories) were assessed by calculating mean (SE) separately for each category.

The questionnaire included four additional questions (see Griffiths et al., 2006 for details): 1) *How personally meaningful was the experience?* (rated from 1 to 8, with 1 = *no more than routine*, and 8 = *the single most meaningful experience of my life*); 2) *Indicate the degree to which the experience was spiritually significant to you?* (rated from 1 to 8, using the same encoding as the first question); 3) *How psychologically challenging were the most psychologically challenging portions of the experiences?* (rated from 1 to 8, using the same encoding as the first question); 4) *How personally psychologically insightful to you were the experiences?* (rated from 1 to 8, using the same encoding as the first question).

### Statistical Analyses

Data analyses and visualizations were performed using the Matplotlib (version 3.2.2), Statsmodels (version 0.11.1), and Pingouin (version 0.3.8) libraries for Python3. A separate multi-linear regression model using the ordinary least square method (OLS) was fit for each outcome variable. The model included *Session* encoded as a dummy independent variable of three levels: baseline, the morning after ceremony, and 7 days after the ceremony. Subsequently, if a main effect of *Session* was found, separate contrasts were performed between baseline, sub-acute, and follow-up sessions. For the MET, if a main effect of *Session* was found on emotional or cognitive empathy, two further analyses were done, separating valence-specific (positive or negative emotion) responses.

Ayahuasca experience ratings were analyzed separately using a one-sample *t-test* comparing the scores on each VAS after ceremony to a zero distribution since previous studies have shown that placebo scores are low, not rising above a 0 in a scale from 0 to 100 (Valle et al., 2016).

Hypothesis-driven correlational analysis was run to try and replicate results from a previous study that showed enhancements in empathic processes were correlated with increased satisfaction with life (Mason et al., 2019). The correlations were run between variables of empathy that showed ayahuasca treatment effects, and satisfaction with life. One-sided Pearson’s correlations were carried out using baseline change scores (24 h after—baseline; 7 days after—baseline). For all statistical analyses, the alpha criterion level of statistical significance was set at *p* ≤ 0.05 and Cohen’s effect (d) size was reported in case of significant results to demonstrate the effect’s magnitude with 0.2–0.5 considered as small, 0.5–0.8 as a medium, and >0.8 as large effect size (Leary, 2014).

## Results

### Participants

**FIGURE 1:**
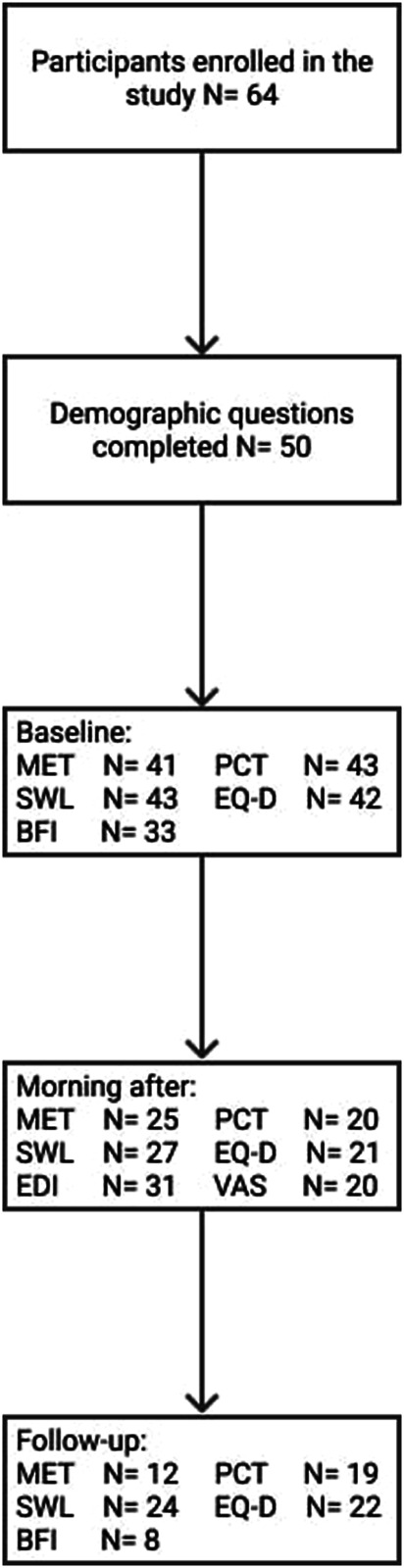
Flow-chart depicting participants’ enrollment.

A total of 64 volunteers signed the informed consent form and agreed to participate in the study. Incomplete or missing test batteries were due to time constraints and/or participant dropout. More information regarding participants’ enrollment and completion rates can be found in Figure 1.

The majority of the participants (55%) were males, 42% were females, and 3% chose not to answer the question. We did not test for possible sex differences at any of the study time points, as it would divide the sample sizes approximately in half, which would result in too small subgroups to conduct meaningful statistics on. The mean (±SD) age of the entire group was 39 ± 11.3 years. Most participants were from Europe (78%), while the rest were from North America (10%), the Middle East (4.7%), or undisclosed (7.3%). The majority of the group (61%) completed an academic level of education. About one-fourth (23.4%) of the participants had used ayahuasca before, and all of them did so during a group retreat. Furthermore, 52% of the participants had previously used psychedelics other than ayahuasca, e.g., psilocybin, LSD, and DMT. When asked about the motivations for attending the ceremony, participants often indicated “to understand myself” (67%), and “to resolve problems” (59%). Next to that, “curiosity” (37.5%) and “other” (31%) motives were mentioned.

### Ayahuasca Sample

**FIGURE 2:**
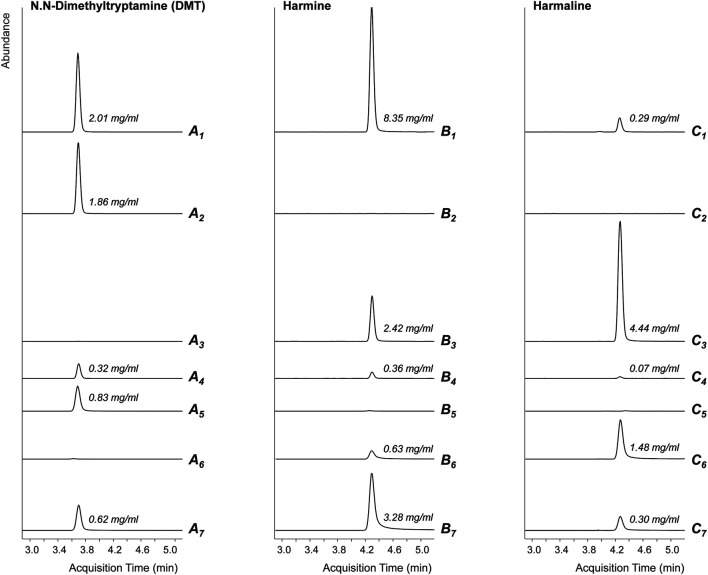
Extracted ion chromatograms of LC-MS analyses of the ayahuasca’s active ingredients DMT **(A)**, harmine **(B)** and harmaline **(C)** in the preparations used in Location 1 (index 1), Location 2 (consisting of Mimosa hostilis, index 2, and Peganum harmala, index 3), Location 3 (index 4) and Location 4 (consisting of Mimosa hostilis, index 5, Peganum harmala, index 6, and a preparation of Banisteriopsis muricata, index 7). The abundances of the analytical signals are equally scaled for each analyte and the concentrations in the brews are annotated.

Table 1 shows the results of the analysis of the ayahuasca samples per organization, the concentrations of which vastly differed between locations; Figure 2 shows the chromatograms of the ayahuasca samples. Based on participants’ reports on the amount of consumed ayahuasca, and metabolites’ analysis afterwards it was calculated that the average (±SD) amount of DMT, harmine, and harmaline was respectively 57.44 (25.77) mg, 127.08 (97.12), and 59.84 (73.27) mg.

**TABLE 1:** Estimated average total dose per site, based on alkaloid concentrations and reported amounts of the brew that participants have taken.

Sample	N from this location	DMT (mg)	Harmine (mg)	Harmaline (mg)
Location 1	15	65.88	251.93	26.25
Location 2	14	69.7	91.00	167.30
Location 3	8	19.20	21.60	4.20
Location 4	3	75.00	143.80	41.60

### Multifaceted Empathy Test

#### Cognitive Empathy

Overall 41 (at baseline), 25 (at the morning after the ceremony), and 12 (at 1 week after the ceremony) participants completed all the parts of MET and were included into analyses. The OLS multi-linear regression revealed a significant main effect of *Session* (F_2,75_ = 12.77; *p* < 0.001) on CE. Compared to baseline, participants correctly identified more emotions the morning after the ayahuasca ceremony (*p* < 0.001; *d* = 0.93), and 1 week after the ceremony (*p* = 0.01; *d* = 0.79; Table 2).

**TABLE 2:** Summary of contrast analyses of the dependent measures.

Variable	Session	N	Mean	SE	*P*	*d*
Total Cognitive Empathy	Baseline	41	16.7	0.88		
	Sub-acute	25	22.67	1.42	<0.001^*^	0.93
	Follow-up	12	21.49	1.84	0.01^*^	0.79
Positive Cognitive Empathy	Baseline	41	8.17	0.51		
	Sub-acute	25	11.12	0.83	<0.001^*^	0.79
	Follow-up	12	11	1.1	0.01^*^	0.8
Negative Cognitive Empathy	Baseline	41	8.54	0.48		
	Sub-acute	25	11.55	0.78	<0.001^*^	0.85
	Follow-up	12	10.49	1.0	0.056	0.59
Average Implicit Emotional Empathy	Baseline	41	4.92	0.21		
	Sub-acute	25	5.3	0.34	0.27	0.25
	Follow-up	12	5.8	0.44	0.047^*^	0.62
Positive Implicit Emotional Empathy	Baseline	41	4.52	0.28		
	Sub-acute	25	4.71	0.46	0.68	0.1
	Follow-up	12	6.05	0.6	0.01^*^	0.78
Negative Implicit Emotional Empathy	Baseline	41	5.32	0.25		
	Sub-acute	25	5.89	0.4	0.15	0.32
	Follow-up	12	5.58	0.5	0.62	0.16
Average Explicit Emotional Empathy	Baseline	41	4.2	0.18		
	Sub-acute	25	4.5	0.29	0.3	0.23
	Follow-up	12	4.37	0.38	0.65	0.14
Convergent Thinking	Baseline	43	10.14	0.42		
	Sub-acute	20	10.55	0.75	0.58	0.13
	Follow-up	19	12.95	0.76	<0.001^*^	0.83
Originality	Baseline	43	5.35	0.72		
	Sub-acute	20	7.9	1.27	0.05	0.52
	Follow-up	19	3.37	1.3	0.13	0.41
Fluency	Baseline	43	4.93	0.65		
	Sub-acute	20	7.5	1.14	0.03	0.55
	Follow-up	19	5.42	0.49	0.68	0.16
Ratio (Originality/Fluency)	Baseline	43	1.23	0.05		
	Sub-acute	20	1.04	0.09	0.04^*^	0.5
	Follow-up	19	0.68	0.1	<0.001^*^	1.27
Satisfaction with Life Scale	Baseline	43	19.1	0.87		
	Sub-acute	27	22.3	1.39	<0.001^*^	1.0
	Follow-up	24	24.9	1.45	<0.001^*^	1.76
Decentering	Baseline	42	3.18	0.07		
	Sub-acute	21	3.76	0.13	<0.001^*^	1.0
	Follow-up	22	4.12	0.13	<0.001^*^	1.76
Personality: *Neuroticism*	Baseline	33	24.58	6.34		
	Follow-up	8	19.5	3.66	0.037^*^	0.98

^*^ *p* < 0.05

When assessing valence-specific responses, analysis revealed a significant main effect of *Session* on recognition of both positive (F_2,75_ = 11.82; *p* < 0.001) and negative (F_2,75_ = 8.47, *p* = 0.005) emotions (Table 2) indicating that the morning after the ceremony, participants correctly recognized more positive and negative emotions than during the baseline measurement (*p* < 0.001; *d* = 0.79 and *p* < 0.001; *d* = 0.85, respectively). At follow-up, participants correctly recognized more positive emotions (*p* = 0.01; *d* = 0.8) compared to baseline; whereas recognition of negative emotions did not differ statistically between baseline and 7-days follow-up (*p* = 0.056; *d* = 0.59).

#### Emotional Empathy

The OLS multi-linear regression model revealed a significant effect of *Session* on Implicit EE (F_2,75_ = 4.42; *p* = 0.04; Table 2). Compared to baseline, participants reported significantly higher arousal levels during the follow-up when confronted with emotional expressions (*p* = 0.047; *d* = 0.62). There was no significant effect of *Session* on implicit EE (*p* = 0.266), when comparing baseline to sub-acute measurement. Furthermore, the one-factor, multi-linear regression model did not reveal a significant main effect of *Session* on the average scores of Explicit EE scores (“concern”, F_2,75_ = 0.572; *p* = 0.452).

Similar time effects were found for valence-specific responses. The analysis revealed an overall effect of *Session* towards positive emotions (F_2,75_ = 5.193; *p* = 0.025; Table 2) and no changes in arousal levels towards negative pictures (F_2,75_ = 0.218; *p* = 0.642). Specifically, compared to baseline, participants reported significantly higher arousal levels towards positive pictures 1 week after the ceremony (*p* = 0.012; *d* = 0.78); these effects were not present at follow-up (*p* = 0.679).

### Picture Concept Test

#### Convergent Thinking

In total 43, 20, and 19 participants completed the first part (convergent thinking) of the PCT during the first, second, and third assessment, respectively. Analysis revealed a significant main effect of *Session* on convergent thinking (F_2,79_ = 12.08; *p* < 0.001; Table 2). Separate contrasts showed that the number of correct solutions non-significantly increased after the ayahuasca ceremony (*p* = 0.58), reaching statistical significance at follow-up measurement (*p* < 0.001; *d* = 0.83).

#### Divergent Thinking

Analysis revealed no effect of *Session* on *originality* (F_2,79_ = 0.858; *p* = 0.357; Table 2), nor *fluency* scores (F_2,79_ = 0.717; *p* = 0.4). When the *originality* scores were weighted for *fluency* (the *ratio*) a significant effect of *Session* was found (F_2,65_ = 30.57; *p* < 0.001), with *ratio*, an estimator of divergent thinking’s quality, being significantly lower the morning after and 1 week after the ceremony than at the baseline (*p* = 0.038; *d* = 0.5 and *p* < 0.001; *d* = 1.27, respectively).

### Satisfaction with Life Scale

The questionnaire was completed by 43 participants at baseline, 27 the morning after the ceremony, and 24 at the follow-up. The multi-linear regression analysis revealed a significant main effect of *Session* on participants’ satisfaction with life ratings (F_2,91_ = 17.0; *p* < 0.001; Table 2). Separate contrasts indicated that, compared to baseline, satisfaction with life significantly increased the morning after (*p* = 0.023; *d* = 0.5) and 1-week after the ceremony (*p* < 0.001; *d* = 0.9).

### Experience Questionnaire-decentering

The questionnaire was completed by 42 participants at the baseline, 21 on the morning after the ceremony, and 22 participants at the follow-up. Analysis showed a significant effect of *Session* on participants’ rating of decentering (F_2,82_ = 60.67; *p* < 0.001; Table 2). Contrasts revealed a significant continuous, linear increase of participants’ decentering scores the morning after (*p*=<0.001; *d* = 1) and 1 week after the ceremony (and *p* < 0.001; *d* = 1.76) compared to baseline.

## The Psychedelic Experience

### Ego Dissolution Inventory

In total, 31 participants filled in the ego dissolution inventory the morning after the ceremony. The mean (SD) of the sample was 45.8 (33.7), with ratings varying between 0 (minimal reported score) and 95.6 (maximal recorded rating).

### Visual Analogue Scale

**FIGURE 3:**
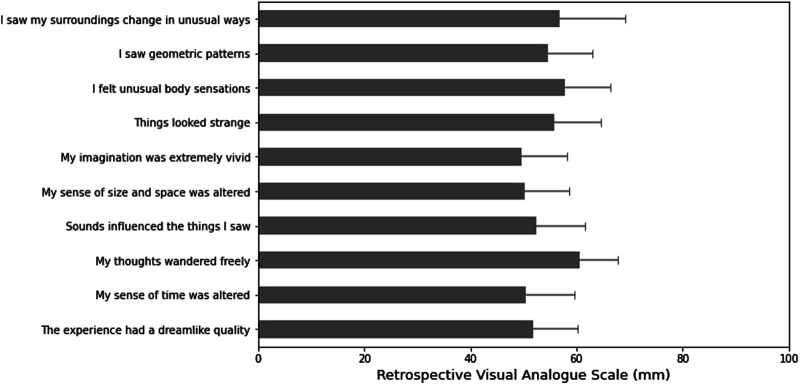
Mean (±SE) of visual analogue scale item scores on a 100 mm scale. Scores are retrospective of the ceremony experience.

Overall, 20 participants filled in the visual analogue scale the morning after the ceremony. Mean (SE) ratings on the different VAS items are shown in Figure 3. The one-sample *t-test* showed ayahuasca-induced significant increases in all VAS items (*t*_19_ = 5.15–7.95; *p* ≤ 0.000; *d* = 1.63–2.51).

### Big Five Inventory

Personality was assessed twice; before (N = 33) and 1 week after the ceremony (N = 8). Linear regression analysis revealed a significant effect of *Session* on participants’ ratings of *neuroticism* (F_1,39_ = 4.68; *p* = 0.037; d = 0.98; Table 2), with scores being significantly lower at the 1-week follow-up than at the baseline. Participants’ reports did not indicate any other significant changes in the remaining personality traits o*penness*, *extraversion*, *agreeableness*, *conscientiousness*) of the BFI scale.

### Persisting Effects Questionnaire

In total, 17 participants filled in the PEQ 7 days after the ceremony. Mean (SE) of the positive and negative ratings assessing attitudes, mood, social effects, and behavior, measured after ayahuasca ceremony by the PEQ are presented in Table 3.

**TABLE 3:** Mean (SE) scores on the first part of the Persisting Effects Questionnaire.

Variable	Positive mean (SE)	Negative mean (SE)
Attitudes about life	57.41 (3.77)	15.71 (0.65)
Attitudes about self	45.06 (3.06)	12.71 (0.43)
Mood changes	38.82 (2.93)	9.47 (0.23)
Social effects	33.00 (3.12)	11.35 (0.78)
Behavior changes	4.41 (0.39)	1.06 (0.06)

On the question, “how personally meaningful was the experience” 2 (11.8%) rated it as the single most meaningful experience of their lives, whereas 9 (52.9%) and 3 (17.6%), rated it as among the 5 and 10 most meaningful experiences of their lives, respectively. One participant (5.9%) rated it as similar to the meaningful experiences that occur on average once every 5 years, one (5.9%) stated it was similar to meaningful experiences that occur once a year, and one (5.9%) said it was similar to experiences that occur on average once a month.

On the question, “how spiritually significant was the experience,” 8 (47.1%) rated it as the most spiritually significant experience of their lives, whereas 4 (23.5%) and 2 (11.8%) rated it as among the 5 and 10 most spiritual experiences of their lives, respectively. One participant (5.9%) rated it as similar to the spiritually meaningful experiences that occur on average once every 5 years, one (5.9%) stated it was similar to spiritually meaningful experiences that occur once a year, and one (5.9%) stated it was similar to spiritually meaningful experiences that occur on average once a month.

In regards to how psychologically challenging the experience was, 2 (11.8%) rated it as the single most difficult or challenging experience of their lives, 6 (35.3%) rated it as among the five most challenging experiences of their lives, and 3 (17.6%) rated it as among the top 10 of most challenging experiences of their lives, followed by 2 (11.8%) stating it was similar to the challenging experiences that occur every 5 years, 2 (11.8%) who said occur every once a year, and 2 (11.8%) who said occur once a month.

Finally, regarding the psychologically insightfulness of the experience, 4 (23.5%) stated the experience to be the single most psychological insightful experience of their lives, 5 (29.4%), and 4 (23.5%) stated among the 5 and 10 most insightful experiences, respectively. One (5.9%) stated that the experience was similar to psychologically insightful experiences that occur on average once every 5 years, two (11.8%) stated it was similar to experiences that occur on average once a month and one (5.9%) stated the experience was no different from every psychologically insightful experience.

### Hypothesis-Driven Correlations

Analysis showed a correlation between changes in implicit EE towards a positive stimulus, and persisting (7 days) changes in satisfaction with life (r = 0.584, *p* = 0.038, n = 10, *one-sided*). Namely, as changes in arousal to pictures of people in positive mood states increased, so did changes in satisfaction with life when comparing baseline to 7 days after intake.

## Discussion

The primary objective of the present study was to complement the existing findings on sub-acute and long-term effects of ayahuasca on flexible cognition, empathy, satisfaction with life, and personality, using a comprehensive test battery with participants attending ayahuasca ceremonies. Relative to the baseline, we observed increases with medium and large effect sizes of convergent thinking (CT), cognitive and implicit emotional empathy (IEE), satisfaction with life (SWLS), and decentering. We also found sub-acute and long-term decreases in the estimator of divergent thinking quality (*ratio*, defined as *originality* weighted for *fluency*). In line with our assumptions, we found decreases in the personality trait *neuroticism* at 1 week post-ceremony, however contrary to our hypotheses no improvements in *openness* were found.

The findings demonstrate post-ayahuasca ceremony increases in participants’ ability to correctly recognize emotional states, for pictures depicting both positive and negative emotions. To our knowledge, this is the first study to demonstrate large, sub-acute, and long-term increases in cognitive empathy among attendees of ayahuasca ceremonies. The present findings contrast with those of previous research with psychedelics and empathogens, where emotional recognition was unaffected (Kuypers et al., 2017; Pokorny et al., 2017; Mason et al., 2019; Uthaug et al., 2021) or even impaired after the administration of LSD, psilocybin, ayahuasca, and MDMA (Bedi et al., 2010; Kometer et al., 2012; Hysek et al., 2014; Dolder et al., 2016). Regarding emotional empathy (EE), a time- and construct-specific differentiation of effects was found, with increases of implicit EE 1 week after the ceremony and no effects on explicit EE. Similarly to the study of Pokorny and colleagues (2017), the present findings show medium increases in participants’ ratings of arousal (implicit EE) towards positive emotions 1 week after the ceremony. Overall, there is growing evidence supporting the claim of acute enhancements in EE after administration of psychedelics and other serotonergic compounds, like MDMA and psilocybin (Hysek et al., 2014; Schmid et al., 2014; Preller et al., 2015; Kuypers et al., 2017; Pokorny et al., 2017; Kuypers, 2018). The current study adds to this body of literature by pointing out the possible long-term enhancements of emotion recognition/awareness, which might bring interesting therapeutic applications of psychedelics’ use for stress-related psychopathologies like depression, anxiety disorders, and post-traumatic stress disorder, which are characterized by low levels of empathy (Donges et al., 2005; Chamberlain et al., 2006; Nietlisbach and Maercker 2009; Cusi et al., 2011; Palm and Follette 2011; Lee and Orsillo 2014; Parlar et al., 2014; Morrison et al., 2016). A hypothesis-driven correlation analysis indeed suggested a positive correlation between sub-acute increases in ratings of emotional empathy and 7-days increases in satisfaction with life; in line with a previous study which found the same relationship after ingestion of psilocybin in a naturalistic setting (Mason et al., 2019). Taken together, results provide limited evidence for directionality, as they demonstrate that an earlier (morning after) increase in positive empathy strongly correlates with a later (7 days after) increase in well-being. However, future research should more formally assess a causal relationship between (positive) empathy and well-being, Additionally, assessments of sleep quality could be considered in future research since sleep quality is related to both constructs, and ayahuasca has been shown to affect sleep parameters, although the behavioral consequences are yet to be examined (Barbanoj et al., 2008).

With regard to convergent thinking (CT), the study showed that at 1-week post-ceremony the performance was significantly increased, which is in line with previous studies showing enhancements in CT at 1 week (Mason et al., 2019), and 1 month (Uthaug et al., 2018) post-psychedelic ceremony, but not the morning after. These findings, although promising, should be interpreted with caution as practice effects cannot be ruled out. To minimize this, parallel versions of the test were used at each assessment point, and no feedback on performance was given. Nevertheless, practice effects have been suggested when utilizing this task in a placebo-controlled, experimental study with psilocybin (Mason et al., 2021).

The current study found decrements in divergent thinking (quantified as the *ratio* of *originality* and *fluency*) at both post-ceremonial assessments. Naturalistic studies that have used this task (Picture Concept Test) have shown mixed results, with one study finding unaffected divergent thinking 1 day after an ayahuasca ceremony (Uthaug et al., 2018), and others pointing out acute (Kuypers et al., 2016) and sub-acute (Mason et al., 2019) increases in DT following respectively the ingestion of ayahuasca and psilocybin in a social setting. Recently, a placebo-controlled study assessing the effect of psilocybin on acute and persisting creative cognition found an acute decrease in deliberate, task-based DT across creativity tasks, and a simultaneous acute increase in ratings of spontaneous creative insight (Mason et al., 2021). Conversely, 7 days later, a DT variable was found to be increased, in that individuals were able to come up with more new ideas for one of the creativity tasks. Findings suggest a time- and test-related differentiation of effects on creative thinking, which should be considered when assessing creativity in future studies (Mason et al., 2021).

Self-rated satisfaction with life (SWL) increased during the sub-acute and long-term assessment when compared to baseline. According to an interpretation of the SWL scores provided by Diener et al. (1985), the participants in the current study, with a mean score of 19 at baseline, can be classified as marginally below average (range 15–19) at the start of the study, indicating small but significant problems in several areas of life or one area that represents a substantial problem (Pavot et al., 1991). During the sub-acute and follow-up assessments, participants’ scores increased up to average (range 20–24) and marginally high (range 25–29) levels of SWL, respectively. Although people with average and high SWL are generally satisfied with their lives, the individuals with average scores desire greater improvements in one or several life’s domains (Pavot et al., 1991). These findings are consistent with the previous studies showing sub-acute (Uthaug et al., 2018; Mason et al., 2019), and long-term (Bouso et al., 2012; Carhart-Harris et al., 2016; Griffiths et al., 2016; Johnson et al., 2017) positive psychological changes after psychedelics use. These findings add to the growing number of empirical evidence indicating that beneficial subjective psychological changes induced by psychedelics outlast the state of acute effects that in case of ayahuasca, last for approximately 4 h (Riba et al., 2001). Although some of the ayahuasca compounds (e.g., THH) were demonstrated to have a half-life of almost 9 h (Callaway et al., 1999), studies employing a combination of electroencephalogram (EEG) recordings and quantification of ayahuasca’s compounds concluded that it seems unlikely that those compounds play any major role in the acute psychoactive effects of ayahuasca (Schenberg et al., 2015).

As hypothesized, the study found decreases in *neuroticism* 1 week after the ayahuasca ceremony; the other four traits were unaffected. The fact that personality can change after a psychedelic experience has been shown previously (Griffiths et al., 2011; MacLean et al., 2011; Bouso et al., 2015; Johnstad 2021), however, most of the studies showed changes in *openness* which were not found in the present study. Previously it has been suggested that neuroticism could lead to difficult experiences when taking a psychedelic (Barrett et al., 2017), though other studies did not show this relation (Studerus et al., 2012; Haijen et al., 2018). Of interest here is that a previous study showed that individuals with low psychological well-being and higher scores of neuroticism report consuming psychedelic substances with the achievement of positive outcomes (Mason et al., 2020). Therefore, psychedelic-related changes in personality traits should be further investigated, to understand how personality changes over time and how this might facilitate the therapeutic process.

In the current study, we also found significant increases in ratings of decentering, both 24 h and 7 days after ingestion of ayahuasca. Findings are in line with previous studies, which havefound increases in decentering ability and interrelated mindfulness capacities 24 h (Soler et al., 2016; Uthaug et al., 2018; Murphy-Beiner and Soar 2020), 15 days (González et al., 2020), and 2 months (Sampedro et al., 2017) post ayahuasca intake. Decentering is described as the ability to take an observer’s perspective on thoughts and emotions (Travers-Hill et al., 2017; Duncan et al., 2021), and is proposed to consist of three processes: meta-awareness, or the explicit awareness of the content of current consciousness, self-distancing from internal experiences (Kross et al., 2005), and reduced reactivity to thought content (Bernstein et al., 2015). Interestingly, previous work has found that when individuals are directed to analyze their feelings about negative autobiographical experiences from a self-distanced, decentered perspective (i.e. visualizing events from the perspective of an observer) versus a self-immersed, first-person perspective, they demonstrate less acute emotional and physiological reactivity, and are less likely to ruminate over time (Kross et al., 2005; Kross and Ayduk 2008). Drawing from this distinction, Ayduk & Kross (2010) have hypothesized that adopting and maintaining a self-distanced perspective while reflecting on emotions allows individuals to reconstruct their feelings and the meaning of their experience in ways that promote insight. This is interesting to think of in light of what individuals report during a psychedelic experience. Namely, individuals report reliving autobiographical memories, often viewed from a distanced, more detached view (Riba et al., 2001; Shanon, 2002; Riba et al., 2006), and subsequently report insights into their problems (Studerus et al., 2010; Roseman et al., 2018; Garcia-Romeu et al., 2019). Given the similarity of these reports, and the finding of enhanced decentering abilities after a psychedelic, the concept of decentering is something that should be investigated more in the framework of psychedelic therapy, as this ability has been suggested to facilitate adaptive coping with depression, stress, and anxiety (Teasdale et al., 2002; Bieling et al., 2012; Hoge et al., 2015; O’Toole et al., 2019; Duncan et al., 2021) and thus drug-induced enhancements of such may play a role in the long-term clinical outcomes being observed in psychedelic clinical trials.

### Limitations and Conclusion

The strength of this study, being naturalistic, is also its greatest limitation. While the ecological validity is high, the sample is self-selected, and it suffers from typically high drop-out rates. However, given the accumulated evidence on the importance of set (mind state and expectations) and setting (physical and social environment) on the outcomes of experience (Frecska et al., 2016; Hartogsohn 2016; Lawn et al., 2018; Sapoznikow et al., 2019), it is important to have an ecologically valid environment with participants enrolling in research, not engaging to receive a reward but to contribute to science. That said, it could also be that highly motivated people with a positive attitude were attracted to participate, hence generalisation towards the general population is difficult for this type of studies. The absence of a control group or placebo makes it challenging to draw hard conclusions, although the fact that the majority of findings (cognitive empathy, satisfaction with life, decentering) were in line with previous studies, some of which were placebo-controlled, is reassuring that the findings might be related to the psychedelic ceremony rather than practice effects or increased familiarity with the test material. Despite the promising character of the present findings, the high heterogeneity of methodological approaches in the psychedelic field (such as the time of assessment, setting, type of the substance) and the sparse number of studies investigating the effects of ayahuasca, with empathy in particular, hampers the formation of comprehensive conclusions. Given that core features of mood disorders include repetitive and rigid patterns of negative and compulsive thoughts, together with social difficulties and impaired empathic abilities (Nietlisbach and Maercker, 2009; Aldao and Nolen-Hoeksema, 2010; Morrison et al., 2016; Santos et al., 2016), the further investigation of the preliminary benefits reported by the current and previous findings is of major importance. For instance, future placebo-controlled experimental studies could investigate potential non-pharmacological influences, while future longitudinal naturalistic research could further investigate the therapeutic potential of psychedelic drugs.

## Data Availability

The original contributions presented in the study are included in the article/Supplementary Material, further inquiries can be directed to the corresponding author.

## References

[B1] Aldao A. Nolen-Hoeksema S. (2010). Specificity of Cognitive Emotion Regulation Strategies: a Transdiagnostic Examination. Behav. Res. Ther. 48 (10), 974–983. 10.1016/j.brat.2010.06.002 20591413

[B2] Ayduk O. Kross E. (2010). From a Distance: Implications of Spontaneous Self-Distancing for Adaptive Self-Reflection. J. Pers Soc. Psychol. 98 (5), 809–829. 10.1037/a0019205 20438226 PMC2881638

[B3] Barbanoj M. J. Riba J. Clos S. Giménez S. Grasa E. Romero S. (2008). Daytime Ayahuasca Administration Modulates REM and Slow-Wave Sleep in Healthy Volunteers. Psychopharmacology (Berl) 196 (2), 315–326. 10.1007/s00213-007-0963-0 18030450

[B4] Barbosa P. C. Giglio J. S. Dalgalarrondo P. (2005). Altered States of Consciousness and Short-Term Psychological After-Effects Induced by the First Time Ritual Use of Ayahuasca in an Urban Context in Brazil. J. Psychoactive Drugs 37 (Issue 2), 193–201. 10.1080/02791072.2005.10399801 16149333

[B5] Barrett F. S. Johnson M. W. Griffiths R. R. (2017). Neuroticism Is Associated with Challenging Experiences with Psilocybin Mushrooms. Pers Individ Dif 117, 155–160. 10.1016/j.paid.2017.06.004 28781400 PMC5540159

[B6] Bedi G. Hyman D. de Wit H. (2010). Is Ecstasy an "empathogen"? Effects of ±3,4-methylenedioxymethamphetamine on Prosocial Feelings and Identification of Emotional States in Others. Biol. Psychiatry 68 (12), 1134–1140. 10.1016/j.biopsych.2010.08.003 20947066 PMC2997873

[B7] Bernstein A. Hadash Y. Lichtash Y. Tanay G. Shepherd K. Fresco D. M. (2015). Decentering and Related Constructs. Perspect. Psychol. Sci. 10 (5), 599–617. 10.1177/1745691615594577 26385999 PMC5103165

[B8] Bieling P. J. Hawley L. L. Bloch R. T. Corcoran K. M. Levitan R. D. Young L. T. (2012). Treatment-specific Changes in Decentering Following Mindfulness-Based Cognitive Therapy versus Antidepressant Medication or Placebo for Prevention of Depressive Relapse. J. Consult Clin. Psychol. 80 (3), 365–372. 10.1037/a0027483 22409641 PMC3365628

[B9] Bouso J. C. González D. Fondevila S. Cutchet M. Fernández X. Ribeiro Barbosa P. C. (2012). Personality, Psychopathology, Life Attitudes and Neuropsychological Performance Among Ritual Users of Ayahuasca: a Longitudinal Study. PLoS One 7 (8), e42421. 10.1371/journal.pone.0042421 22905130 PMC3414465

[B10] Bouso J. C. Palhano-Fontes F. Rodríguez-Fornells A. Ribeiro S. Sanches R. Crippa J. A. (2015). Long-term Use of Psychedelic Drugs Is Associated with Differences in Brain Structure and Personality in Humans. Eur. Neuropsychopharmacol. 25 (4), 483–492. 10.1016/j.euroneuro.2015.01.008 25637267

[B11] Callaway J. C. McKenna D. J. Grob C. S. Brito G. S. Raymon L. P. Poland R. E. (1999). Pharmacokinetics of Hoasca Alkaloids in Healthy Humans. J. Ethnopharmacol 65 (3), 243–256. 10.1016/s0378-8741(98)00168-8 10404423

[B12] Carhart-Harris R. L. Bolstridge M. Rucker J. Day C. M. Erritzoe D. Kaelen M. (2016). Psilocybin with Psychological Support for Treatment-Resistant Depression: an Open-Label Feasibility Study. Lancet Psychiatry 3 (7), 619–627. 10.1016/s2215-0366(16)30065-7 27210031

[B13] Carhart-Harris R. L. Erritzoe D. Williams T. Stone J. M. Reed L. J. Colasanti A. (2012). Neural Correlates of the Psychedelic State as Determined by fMRI Studies with Psilocybin. Proc. Natl. Acad. Sci. U S A. 109 (6), 2138–2143. 10.1073/pnas.1119598109 22308440 PMC3277566

[B14] Chamberlain S. R. Fineberg N. A. Blackwell A. D. Robbins T. W. Sahakian B. J. (2006). Motor Inhibition and Cognitive Flexibility in Obsessive-Compulsive Disorder and Trichotillomania. Am. J. Psychiatry 163 (7), 1282–1284. 10.1176/appi.ajp.163.7.1282 16816237

[B15] Cusi A. M. Macqueen G. M. Spreng R. N. McKinnon M. C. (2011). Altered Empathic Responding in Major Depressive Disorder: Relation to Symptom Severity, Illness burden, and Psychosocial Outcome. Psychiatry Res. 188 (2), 231–236. 10.1016/j.psychres.2011.04.013 21592584

[B16] Da Silveira D. X. Grob C. S. de Rios M. D. Lopez E. Alonso L. K. Tacla C. (2005). Ayahuasca in Adolescence: a Preliminary Psychiatric Assessment. J. Psychoactive Drugs 37 (2), 129–133. 10.1080/02791072.2005.10399792 16149324

[B17] de Lima Osório F. de Macedo L. R. H. de Sousa J. P. M. Pinto J. P. Quevedo J. de Souza Crippa J. A. (2011). The Therapeutic Potential of Harmine and Ayahuasca in Depression: Evidence from Exploratory Animal and Human Studies. The Ethnopharmacology of Ayahuasca 75, 85.

[B18] Diener E. Emmons R. A. Larsen R. J. Griffin S. (1985). The Satisfaction with Life Scale. J. Pers Assess. 49 (Issue 1), 71–75. 10.1207/s15327752jpa4901_13 16367493

[B19] Dolder P. C. Schmid Y. Müller F. Borgwardt S. Liechti M. E. (2016). LSD Acutely Impairs Fear Recognition and Enhances Emotional Empathy and Sociality. Neuropsychopharmacology 41 (11), 2638–2646. 10.1038/npp.2016.82 27249781 PMC5026740

[B20] Domínguez-Clavé E. Soler J. Pascual J. C. Elices M. Franquesa A. Valle M. (2019). Ayahuasca Improves Emotion Dysregulation in a Community Sample and in Individuals with Borderline-like Traits. Psychopharmacology (Berl) 236 (2), 573–580. 10.1007/s00213-018-5085-3 30406413

[B21] Donges U. S. Kersting A. Dannlowski U. Lalee-Mentzel J. Arolt V. Suslow T. (2005). Reduced Awareness of Others' Emotions in Unipolar Depressed Patients. J. Nerv Ment. Dis. 193 (5), 331–337. 10.1097/01.nmd.0000161683.02482.19 15870617

[B22] dos Santos R. G. Osório F. L. Crippa J. A. S. Riba J. Zuardi A. W. Hallak J. E. C. (2016). Antidepressive, Anxiolytic, and Antiaddictive Effects of Ayahuasca, Psilocybin and Lysergic Acid Diethylamide (LSD): a Systematic Review of Clinical Trials Published in the Last 25 Years. Ther. Adv. Psychopharmacol. 6 (3), 193–213. 10.1177/2045125316638008 27354908 PMC4910400

[B23] Duncan N. S. Zimmer-Gembeck M. J. Gardner A. A. Modecki K. (2021). The Measurement and Benefit of Decentering for Coping Self-Efficacy, Flexibility, and Ways of Coping with Interpersonal Stress. Personal. Individual Differences 179, 110932. 10.1016/j.paid.2021.110932

[B24] Dziobek I. Rogers K. Fleck S. Bahnemann M. Heekeren H. R. Wolf O. T. (2008). Dissociation of Cognitive and Emotional Empathy in Adults with Asperger Syndrome Using the Multifaceted Empathy Test (MET). J. Autism Dev. Disord. 38 (3), 464–473. 10.1007/s10803-007-0486-x 17990089

[B25] Erritzoe D. Roseman L. Nour M. M. MacLean K. Kaelen M. Nutt D. J. (2018). Effects of Psilocybin Therapy on Personality Structure. Acta Psychiatr. Scand. 138 (5), 368–378. 10.1111/acps.12904 29923178 PMC6220878

[B26] Frecska E. Bokor P. Winkelman M. (2016). The Therapeutic Potentials of Ayahuasca: Possible Effects against Various Diseases of Civilization. Front. Pharmacol. 7, 35. 10.3389/fphar.2016.00035 26973523 PMC4773875

[B27] Frecska E. Móré C. E. Vargha A. Luna L. E. (2012). Enhancement of Creative Expression and Entoptic Phenomena as After-Effects of Repeated Ayahuasca Ceremonies. J. Psychoactive Drugs 44 (3), 191–199. 10.1080/02791072.2012.703099 23061318

[B28] Fresco D. M. Moore M. T. van Dulmen M. H. Segal Z. V. Ma S. H. Teasdale J. D. (2007). Initial Psychometric Properties of the Experiences Questionnaire: Validation of a Self-Report Measure of Decentering. Behav. Ther. 38 (3), 234–246. 10.1016/j.beth.2006.08.003 17697849

[B29] Garcia-Romeu A. Davis A. K. Erowid F. Erowid E. Griffiths R. R. Johnson M. W. (2019). Cessation and Reduction in Alcohol Consumption and Misuse after Psychedelic Use. J. Psychopharmacol. 33 (9), 1088–1101. 10.1177/0269881119845793 31084460

[B30] González D. Cantillo J. Pérez I. Farré M. Feilding A. Obiols J. E. (2020). Therapeutic Potential of Ayahuasca in Grief: a Prospective, Observational Study. Psychopharmacology (Berl) 237 (4), 1171–1182. 10.1007/s00213-019-05446-2 31938878 PMC7113212

[B31] Griffiths R. R. Johnson M. W. Carducci M. A. Umbricht A. Richards W. A. Richards B. D. (2016). Psilocybin Produces Substantial and Sustained Decreases in Depression and Anxiety in Patients with Life-Threatening Cancer: A Randomized Double-Blind Trial. J. Psychopharmacol. 30 (12), 1181–1197. 10.1177/0269881116675513 27909165 PMC5367557

[B32] Griffiths R. R. Johnson M. W. Richards W. A. Richards B. D. McCann U. Jesse R. (2011). Psilocybin Occasioned Mystical-type Experiences: Immediate and Persisting Dose-Related Effects. Psychopharmacology (Berl) 218 (4), 649–665. 10.1007/s00213-011-2358-5 21674151 PMC3308357

[B33] Griffiths R. R. Richards W. A. McCann U. Jesse R. (2006). Psilocybin Can Occasion Mystical-type Experiences Having Substantial and Sustained Personal Meaning and Spiritual Significance. Psychopharmacology (Berl) 187 (3), 268–292. 10.1007/s00213-006-0457-5 16826400

[B34] Haijen E. C. H. M. Kaelen M. Roseman L. Timmermann C. Kettner H. Russ S. (2018). Predicting Responses to Psychedelics: A Prospective Study. Front. Pharmacol. 9. 10.3389/fphar.2018.00897 PMC6225734 30450045

[B35] Hartogsohn I. (2016). Set and Setting, Psychedelics and the Placebo Response: An Extra-pharmacological Perspective on Psychopharmacology. J. Psychopharmacol. 30 (12), 1259–1267. 10.1177/0269881116677852 27852960

[B36] Hoge E. A. Bui E. Goetter E. Robinaugh D. J. Ojserkis R. A. Fresco D. M. (2015). Change in Decentering Mediates Improvement in Anxiety in Mindfulness-Based Stress Reduction for Generalized Anxiety Disorder. Cogn. Ther. Res. 39 (Issue 2), 228–235. 10.1007/s10608-014-9646-4 PMC5354303 28316355

[B37] Hysek C. M. Simmler L. D. Schillinger N. Meyer N. Schmid Y. Donzelli M. (2014). Pharmacokinetic and Pharmacodynamic Effects of Methylphenidate and MDMA Administered Alone or in Combination. Int. J. Neuropsychopharmacol. 17 (3), 371–381. 10.1017/S1461145713001132 24103254

[B38] John O. P. Donahue E. M. Kentle R. L. (1991). The Big Five Inventory - Versions 4a and 54. Berkeley: University of California, Institute of Personality and Social Research.

[B39] John O. P. Srivastava S. (1999). “The Big-Five Trait Taxonomy: History, Measurement, and Theoretical Perspectives,” Handbook of Personality: Theory and Research. Editors Pervin L. A. John O. P., (New York: Guilford Press) Vol. 2, 102–138.

[B40] Johnson M. W. Garcia-Romeu A. Griffiths R. R. (2017). Long-term Follow-Up of Psilocybin-Facilitated Smoking Cessation. Am. J. Drug Alcohol. Abuse 43 (Issue 1), 55–60. 10.3109/00952990.2016.1170135 27441452 PMC5641975

[B41] Johnstad P. G. (2021). The Psychedelic Personality: Personality Structure and Associations in a Sample of Psychedelics Users. J. Psychoactive Drugs 53 (2), 97–103. 10.1080/02791072.2020.1842569 33252034

[B42] Kometer M. Schmidt A. Bachmann R. Studerus E. Seifritz E. Vollenweider F. X. (2012). Psilocybin Biases Facial Recognition, Goal-Directed Behavior, and Mood State toward Positive Relative to Negative Emotions through Different Serotonergic Subreceptors. Biol. Psychiatry 72 (11), 898–906. 10.1016/j.biopsych.2012.04.005 22578254

[B43] Kross E. Ayduk O. (2008). Facilitating Adaptive Emotional Analysis: Distinguishing Distanced-Analysis of Depressive Experiences from Immersed-Analysis and Distraction. Pers Soc. Psychol. Bull. 34 (7), 924–938. 10.1177/0146167208315938 18469151

[B44] Kross E. Ayduk O. Mischel W. (2005). When Asking "why" Does Not Hurt. Distinguishing Rumination from Reflective Processing of Negative Emotions. Psychol. Sci. 16 (9), 709–715. 10.1111/j.1467-9280.2005.01600.x 16137257

[B45] Kuypers K. P. Riba J. de la Fuente Revenga M. Barker S. Theunissen E. L. Ramaekers J. G. (2016). Ayahuasca Enhances Creative Divergent Thinking while Decreasing Conventional Convergent Thinking. Psychopharmacology (Berl) 233 (18), 3395–3403. 10.1007/s00213-016-4377-8 27435062 PMC4989012

[B46] Kuypers K. P. C. Dolder P. C. Ramaekers J. G. Liechti M. E. (2017). Multifaceted Empathy of Healthy Volunteers after Single Doses of MDMA: A Pooled Sample of Placebo-Controlled Studies. J. Psychopharmacol. 31 (5), 589–598. 10.1177/0269881117699617 28372480 PMC5418931

[B47] Kuypers K. P. C. (2018). Out of the Box: A Psychedelic Model to Study the Creative Mind. Med. Hypotheses 115, 13–16. 10.1016/j.mehy.2018.03.010 29685188

[B48] Lawn W. Hallak J. E. Crippa J. A. Dos Santos R. Porffy L. Barratt M. J. (2018). Author Correction: Well-Being, Problematic Alcohol Consumption and Acute Subjective Drug Effects in Past-Year Ayahuasca Users: a Large, International, Self-Selecting Online Survey. Sci. Rep. 8 (1), 4059. 10.1038/s41598-018-21666-6 29497055 PMC5832875

[B49] Leary M. R. (2014). Introduction to Behavioral Research Methods, Vol. 6. Pearson Education Limited.

[B50] Lee J. K. Orsillo S. M. (2014). Investigating Cognitive Flexibility as a Potential Mechanism of Mindfulness in Generalized Anxiety Disorder. J. Behav. Ther. Exp. Psychiatry 45 (1), 208–216. 10.1016/j.jbtep.2013.10.008 24239587

[B51] MacLean K. A. Johnson M. W. Griffiths R. R. (2011). Mystical Experiences Occasioned by the Hallucinogen Psilocybin lead to Increases in the Personality Domain of Openness. J. Psychopharmacol. 25 (11), 1453–1461. 10.1177/0269881111420188 21956378 PMC3537171

[B52] Mason N. L. Kuypers K. P. C. Reckweg J. T. Müller F. Tse D. H. Y. Da Rios B. (2021). Spontaneous and Deliberate Creative Cognition during and after Psilocybin Exposure. Transl Psychiatry 11 (1), 209. 10.1038/s41398-021-01335-5 33833225 PMC8032715

[B53] Mason N. L. Mischler E. Uthaug M. V. Kuypers K. P. C. (2019). Sub-Acute Effects of Psilocybin on Empathy, Creative Thinking, and Subjective Well-Being. J. Psychoactive Drugs 51 (2), 123–134. 10.1080/02791072.2019.1580804 30905276

[B54] Mason N. L. Dolder P. C. Kuypers K. P. (2020). Reported Effects of Psychedelic Use on Those with Low Well-Being Given Various Emotional States and Social Contexts. Drug Sci. Pol. L. 6, 205032451990006. 10.1177/2050324519900068

[B55] Mason N. L. Kuypers K. P. C. (2021). “Acute and Long-Term Effects of Ayahuasca on (Higher-Order) Cognitive Processes,” in Ayahuasca Healing and Science. Editors Labate B. C. Cavnar C. (Springer Nature), 117–136. 10.1007/978-3-030-55688-4_7

[B56] Morrison A. S. Mateen M. A. Brozovich F. A. Zaki J. Goldin P. R. Heimberg R. G. (2016). Empathy for Positive and Negative Emotions in Social Anxiety Disorder. Behav. Res. Ther. 87, 232–242. 10.1016/j.brat.2016.10.005 27816799 PMC5142818

[B57] Murphy-Beiner A. Soar K. (2020). Ayahuasca's 'afterglow': Improved Mindfulness and Cognitive Flexibility in Ayahuasca Drinkers. Psychopharmacology (Berl) 237 (4), 1161–1169. 10.1007/s00213-019-05445-3 31927605

[B58] Nietlisbach G. Maercker A. (2009). Social Cognition and Interpersonal Impairments in Trauma Survivors with PTSD. J. Aggression, Maltreat. Trauma 18 (4), 382–402. 10.1080/10926770902881489

[B59] Nour M. M. Evans L. Carhart-Harris R. L. (2017). Psychedelics, Personality and Political Perspectives. J. Psychoactive Drugs 49 (3), 182–191. 10.1080/02791072.2017.1312643 28443703

[B60] Nour M. M. Evans L. Nutt D. Carhart-Harris R. L. (2016). Ego-Dissolution and Psychedelics: Validation of the Ego-Dissolution Inventory (EDI). Front. Hum. Neurosci. 10, 269. 10.3389/fnhum.2016.00269 27378878 PMC4906025

[B61] O’Toole M. S. Renna M. E. Mennin D. S. Fresco D. M. (2019). Changes in Decentering and Reappraisal Temporally Precede Symptom Reduction during Emotion Regulation Therapy for Generalized Anxiety Disorder with and without Co-occurring Depression. Behav. Ther. 50 (6), 1042–1052. 10.1016/j.beth.2018.12.005 31735240 PMC7441462

[B62] Palhano-Fontes F. Andrade K. C. Tofoli L. F. Santos A. C. Crippa J. A. Hallak J. E. (2015). The Psychedelic State Induced by Ayahuasca Modulates the Activity and Connectivity of the Default Mode Network. PLOS ONE 10 (2), e0118143. 10.1371/journal.pone.0118143 25693169 PMC4334486

[B63] Palhano-Fontes F. Barreto D. Onias H. Andrade K. C. Novaes M. M. Pessoa J. A. (2019). Rapid Antidepressant Effects of the Psychedelic Ayahuasca in Treatment-Resistant Depression: a Randomized Placebo-Controlled Trial. Psychol. Med. 49 (4), 655–663. 10.1017/S0033291718001356 29903051 PMC6378413

[B64] Palhano-Fontes F. Mota-Rolim S. Lobão-Soares B. Galvão-Coelho N. Maia-Oliveira J. P. Araújo D. B. (2021). Recent Evidence on the Antidepressant Effects of Ayahuasca. Ayahuasca Healing Sci. 21. 10.1007/978-3-030-55688-4_2

[B65] Palm K. M. Follette V. M. (2011). The Roles of Cognitive Flexibility and Experiential Avoidance in Explaining Psychological Distress in Survivors of Interpersonal Victimization. J. Psychopathol Behav. Assess. 33 (1), 79–86. 10.1007/s10862-010-9201-x

[B66] Parlar M. Frewen P. Nazarov A. Oremus C. MacQueen G. Lanius R. (2014). Alterations in Empathic Responding Among Women with Posttraumatic Stress Disorder Associated with Childhood Trauma. Brain Behav. 4 (3), 381–389. 10.1002/brb3.215 24944867 PMC4055188

[B67] Pavot W. Diener E. Colvin C. R. Sandvik E. (1991). Further Validation of the Satisfaction with Life Scale: Evidence for the Cross-Method Convergence of Well-Being Measures. J. Pers Assess. 57 (1), 149–161. 10.1207/s15327752jpa5701_17 1920028

[B68] Pokorny T. Preller K. H. Kometer M. Dziobek I. Vollenweider F. X. (2017). Effect of Psilocybin on Empathy and Moral Decision-Making. Int. J. Neuropsychopharmacol. 20 (9), 747–757. 10.1093/ijnp/pyx047 28637246 PMC5581487

[B69] Preller K. H. Pokorny T. Krähenmann R. Dziobek I. Stämpfli P. Vollenweider F. X. (2015). The Effect of 5-HT2A/1a Agonist Treatment on Social Cognition, Empathy, and Social Decision-Making. Eur. Psychiatry J. Assoc. Eur. Psychiatrists 30 (S1), 1. 10.1016/S0924-9338(15)30017-1

[B70] Riba J. Rodríguez-Fornells A. Urbano G. Morte A. Antonijoan R. Montero M. (2001). Subjective Effects and Tolerability of the South American Psychoactive Beverage Ayahuasca in Healthy Volunteers. Psychopharmacology (Berl) 154 (1), 85–95. 10.1007/s002130000606 11292011

[B71] Riba J. Romero S. Grasa E. Mena E. Carrió I. Barbanoj M. J. (2006). Increased Frontal and Paralimbic Activation Following Ayahuasca, the Pan-Amazonian Inebriant. Psychopharmacology (Berl) 186 (1), 93–98. 10.1007/s00213-006-0358-7 16575552

[B72] Riba J. Valle M. Urbano G. Yritia M. Morte A. Barbanoj M. J. (2003). Human Pharmacology of Ayahuasca: Subjective and Cardiovascular Effects, Monoamine Metabolite Excretion, and Pharmacokinetics. J. Pharmacol. Exp. Ther. 306 (1), 73–83. 10.1124/jpet.103.049882 12660312

[B73] Roseman L. Nutt D. J. Carhart-Harris R. L. (2018). Quality of Acute Psychedelic Experience Predicts Therapeutic Efficacy of Psilocybin for Treatment-Resistant Depression. Front. Pharmacol. 8. 10.3389/fphar.2017.00974 PMC5776504 29387009

[B74] Sampedro F. de la Fuente Revenga M. Valle M. Roberto N. Domínguez-Clavé E. Elices M. (2017). Assessing the Psychedelic "After-Glow" in Ayahuasca Users: Post-Acute Neurometabolic and Functional Connectivity Changes Are Associated with Enhanced Mindfulness Capacities. Int. J. Neuropsychopharmacol. 20 (9), 698–711. 10.1093/ijnp/pyx036 28525587 PMC5581489

[B75] Sanches R. F. de Lima Osório F. dos Santos R. G. Macedo L. R. Maia-de-Oliveira J. P. Wichert-Ana L. (2016). Antidepressant Effects of a Single Dose of Ayahuasca in Patients with Recurrent Depression: A SPECT Study. J. Clin. Psychopharmacol. 36 (1), 77–81. 10.1097/jcp.0000000000000436 26650973

[B76] Sapoznikow A. Walsh Z. Tupper K. W. Erowid E. Erowid F. (2019). The Influence of Context on Ayahuasca Experiences: An Analysis of Experience Reports. J. Psychedelic Stud. 3 (3), 288–294. 10.1556/2054.2019.028

[B77] Sarris J. Perkins D. Cribb L. Schubert V. Opaleye E. Bouso J. C. (2021). Ayahuasca Use and Reported Effects on Depression and Anxiety Symptoms: An International Cross-Sectional Study of 11,912 Consumers. J. Affective Disord. Rep. 4, 100098. 10.1016/j.jadr.2021.100098

[B78] Schenberg E. E. Alexandre J. F. Filev R. Cravo A. M. Sato J. R. Muthukumaraswamy S. D. (2015). Acute Biphasic Effects of Ayahuasca. PLoS One 10 (9), e0137202. 10.1371/journal.pone.0137202 26421727 PMC4589238

[B79] Schmid J. T. (2012). The Myth of Ayahuasca. TYPES, EFFICACY AND MYTHS 127. https://www.academia.edu/download/36512865/978-1-61470-657-1_eBook_NOVA.pdf#page=139.

[B80] Schmid Y. Hysek C. M. Simmler L. D. Crockett M. J. Quednow B. B. Liechti M. E. (2014). Differential Effects of MDMA and Methylphenidate on Social Cognition. J. Psychopharmacol. 28 (9), 847–856. 10.1177/0269881114542454 25052243

[B81] Schmid Y. Liechti M. E. (2018). Long-lasting Subjective Effects of LSD in normal Subjects. Psychopharmacology (Berl) 235 (2), 535–545. 10.1007/s00213-017-4733-3 28918441 PMC5813062

[B82] Schultes R. E. (1986). Recognition of Variability in Wild Plants by Indians of the Northwest Amazon: An enigma. J. Ethnobiol. 6 (2). Available at: https://ethnobiology.org/sites/default/files/pdfs/JoE/6-2/Schultes1986.pdf .

[B83] Shanon B. (2002). The Antipodes of the Mind: Charting the Phenomenology of the Ayahuasca Experience. Oxford University Press. https://play.google.com/store/books/details?id=rIhdbLq-Mc0C.

[B84] Soler J. Elices M. Franquesa A. Barker S. Friedlander P. Feilding A. (2016). Exploring the Therapeutic Potential of Ayahuasca: Acute Intake Increases Mindfulness-Related Capacities. Psychopharmacology (Berl) 233 (5), 823–829. 10.1007/s00213-015-4162-0 26612618

[B85] Soler J. Franquesa A. Feliu-Soler A. Cebolla A. García-Campayo J. Tejedor R. (2014). Assessing Decentering: Validation, Psychometric Properties, and Clinical Usefulness of the Experiences Questionnaire in a Spanish Sample. Behav. Ther. 45 (6), 863–871. 10.1016/j.beth.2014.05.004 25311294

[B86] Studerus E. Gamma A. Kometer M. Vollenweider F. X. (2012). Prediction of Psilocybin Response in Healthy Volunteers. PLoS One 7 (2), e30800. 10.1371/journal.pone.0030800 22363492 PMC3281871

[B87] Studerus E. Gamma A. Vollenweider F. X. (2010). Psychometric Evaluation of the Altered States of Consciousness Rating Scale (OAV). PLoS ONE 5 (8), e12412. 10.1371/journal.pone.0012412 20824211 PMC2930851

[B88] Teasdale J. D. Moore R. G. Hayhurst H. Pope M. Williams S. Segal Z. V. (2002). Metacognitive Awareness and Prevention of Relapse in Depression: Empirical Evidence. J. Consult Clin. Psychol. 70 (2), 275–287. 10.1037/0022-006x.70.2.275 11952186

[B89] Travers-Hill E. Dunn B. D. Hoppitt L. Hitchcock C. Dalgleish T. (2017). Beneficial Effects of Training in Self-Distancing and Perspective Broadening for People with a History of Recurrent Depression. Behav. Res. Ther. 95, 19–28. 10.1016/j.brat.2017.05.008 28525796 PMC6614041

[B90] Tull M. T. Roemer L. (2007). Emotion Regulation Difficulties Associated with the Experience of Uncued Panic Attacks: Evidence of Experiential Avoidance, Emotional Nonacceptance, and Decreased Emotional Clarity. Behav. Ther. 38 (4), 378–391. 10.1016/j.beth.2006.10.006 18021952

[B91] Tupper K. W. (2008). The Globalization of Ayahuasca: Harm Reduction or Benefit Maximization. Int. J. Drug Pol. 19 (4), 297–303. 10.1016/j.drugpo.2006.11.001 18638702

[B92] Uthaug M. V. Lancelotta R. van Oorsouw K. Kuypers K. P. C. Mason N. Rak J. (2019). A Single Inhalation of Vapor from Dried Toad Secretion Containing 5-Methoxy-N,N-Dimethyltryptamine (5-MeO-DMT) in a Naturalistic Setting Is Related to Sustained Enhancement of Satisfaction with Life, Mindfulness-Related Capacities, and a Decrement of Psychopathological Symptoms. Psychopharmacology (Berl) 236 (9), 2653–2666. 10.1007/s00213-019-05236-w 30982127 PMC6695371

[B93] Uthaug M. V. van Oorsouw K. Kuypers K. P. C. van Boxtel M. Broers N. J. Mason N. L. (2018). Sub-acute and Long-Term Effects of Ayahuasca on Affect and Cognitive Thinking Style and Their Association with Ego Dissolution. Psychopharmacology (Berl) 235 (10), 2979–2989. 10.1007/s00213-018-4988-3 30105399 PMC6182612

[B94] Uthaug M. V. Mason N. L. Toennes S. W. Reckweg J. T. de Sousa Fernandes Perna E. B. Kuypers K. P. C. (2021). A Placebo-Controlled Study of the Effects of Ayahuasca, Set and Setting on Mental Health of Participants in Ayahuasca Group Retreats. Psychopharmacology 238, 1899–1910. 10.1007/s00213-021-05817-8 33694031 PMC8233273

[B95] Valle M. Maqueda A. E. Rabella M. Rodríguez-Pujadas A. Antonijoan R. M. Romero S. (2016). Inhibition of Alpha Oscillations through serotonin-2A Receptor Activation Underlies the Visual Effects of Ayahuasca in Humans. Eur. Neuropsychopharmacol. 26 (7), 1161–1175. 10.1016/j.euroneuro.2016.03.012 27039035

[B96] van Oorsouw K. I. Uthaug M. V. Mason N. L. Broers N. J. Ramaekers J. G. (2021). Sub-acute and Long-Term Effects of Ayahuasca on Mental Health and Well-Being in Healthy Ceremony Attendants: A Replication Study. J. Psychedelic Stud. 10.1556/2054.2021.00174

[B97] Winkelman M. (2005). Drug Tourism or Spiritual Healing? Ayahuasca Seekers in Amazonia. J. Psychoactive Drugs 37 (2), 209–218. 10.1080/02791072.2005.10399803 16149335

